# Evaluating Word Embedding Feature Extraction Techniques for Host-Based Intrusion Detection Systems

**DOI:** 10.1007/s44248-023-00002-y

**Published:** 2023-03-30

**Authors:** Paul K. Mvula, Paula Branco, Guy-Vincent Jourdan, Herna L. Viktor

**Affiliations:** School of Electrical Engineering and Computer Science (EECS), University of Ottawa, 800 King Edward Avenue, Ottawa, K1N 6N5 ON Canada

**Keywords:** Word embedding, Feature extraction, Cyber-security, Intrusion detection, Syscall traces

## Abstract

Research into Intrusion and Anomaly Detectors at the Host level typically pays much attention to extracting attributes from system call traces. These include window-based, Hidden Markov Models, and sequence-model-based attributes. Recently, several works have been focusing on sequence-model-based feature extractors, specifically Word2Vec and GloVe, to extract embeddings from the system call traces due to their ability to capture semantic relationships among system calls. However, due to the nature of the data, these extractors introduce inconsistencies in the extracted features, causing the Machine Learning models built on them to yield inaccurate and potentially misleading results. In this paper, we first highlight the research challenges posed by these extractors. Then, we conduct experiments with new feature sets assessing their suitability to address the detected issues. Our experiments show that Word2Vec is prone to introducing more duplicated samples than GloVe. Regarding the solutions proposed, we found that concatenating the embedding vectors generated by Word2Vec and GloVe yields the overall best balanced accuracy. In addition to resolving the challenge of data leakage, this approach enables an improvement in performance relative to other alternatives.

## Introduction

Host-based Intrusion Detection Systems (HIDS) may be used to assist organizations in identifying threats within the network perimeter by monitoring host devices for malicious activities that could result in massive breaches if left unchecked [1]. A HIDS based on Machine Learning (ML) methods analyzes data in the form of logs, file systems, connections, or kernel (system) calls, which can be seen as an ordered sequence of system calls that a process performs during its execution. Kernel or system call (syscall) traces are specific to different processes or applications and of arbitrary length, and most ML/DL methods require fixed feature vectors as input for training and detection. Therefore the system call traces must be preprocessed and standardized to a fixed length before they are fed to the ML algorithm for training and detection.

Speed and reliability matter in cyber-security, thus the feature extraction technique used must standardize the syscall trace as fast as possible without losing information before it is sent to the ML algorithm for classification. There exist several feature extraction techniques for system call traces, each with its advantages and peculiarities, but they can be divided into window-based or short-sequence, frequency-based, Hidden Markov Models (HMM) and sequence model-based features. In this research, we introduce the common feature extraction and dimensionality reduction techniques used for standardizing system call traces then evaluate approaches based on Natural Language Processing (NLP). We focus on sequence model-based extractors because they produce embeddings with implicit relationships that are effective for training on data that can benefit from contextual information. It has been demonstrated that those embeddings improve generalization and performance for HIDS, particularly when there is a lack of training data.

Nevertheless, since we are dealing with sequences of numbers and not text, these feature extraction techniques introduce several duplicates which in the worst case appear both in the training and testing sets. A possible solution would be removing the duplicated samples, however, this results in a reduction in variety and the number of samples in the datasets. Moreover, our findings show that in addition to the reduction in diversity and the number of training and testing data, the ML model’s performance is also negatively affected when duplicated vectors are deleted from the dataset prior to building the intrusion detection model. We, therefore, propose additional methods to prevent the performance decline and loss of data diversity, while ensuring that no data leakage occurs.

Below is a summary of our contributions:

1. We show the discrepancies and pitfalls of utilizing word embeddings as features for HIDS.
2. We propose the incorporation of new feature sets to mitigate the research challenges introduced by these feature extraction techniques.
3. We assess the performance of the new feature sets by conducting an extensive set of experiments and we analyze the results using statistical tests.
4. We provide a set of recommendations on the use of the proposed alternate feature sets.

The remainder of the paper is structured as follows. We begin by introducing the different feature extraction methods used for system call traces in Sect. 2. In Sect. 3, we present the research challenges that word-embedding feature extractors introduce in the data and in Sect. 4, we conduct several experiments using alternative feature sets. Section 5 compares and analyzes the results of the additional feature sets followed by Sect. 6, which concludes the work.

## Related Work

Since Forrest [2] stated that system calls may be utilized to detect computer system anomalies two decades ago, several implementations of ML-based HIDS from system call traces, each using different feature extractors or representations have been proposed. Correspondingly, several benchmark datasets for evaluating syscall-based HIDS have been evaluated in the plethora of studies presented over the past few decades.

Earlier works used short-sequence or window-based feature extractors to extract fixed or variable-length windows from the system calls which are used as feature vectors. Sliding-window and *n*-gram feature representations may be seen as window-based features. An *n*-gram is a contiguous sequence of *n* system calls and a sliding window extracts *n*-grams from a system-call trace at different time steps. Depending on the context there can either be a single, fixed, window size or multiple window sizes as the optimal window size is determined by the syscalls in the modelled sub-sequence [3–5]. The short-sequence-based feature extractors tend to be computationally expensive [6]. In contrast, frequency-based feature extractors are computationally cheaper than window-based algorithms because they rearrange system call traces into equal-sized vectors based on the idea of “frequency” and deal exclusively with the resulting frequency vectors [7–11].

A Hidden Markov Model (HMM) is a doubly embedded stochastic process that incorporates one underlying stochastic process embedded within another set of stochastic processes that generate the observations. Although powerful, HMMs are known for often being computationally expensive, having larger storage requirements, especially when syscall traces consist of several calls, and yielding sub-optimal accuracy when constructing subject behaviour [12]. They also require hyper-parameter tuning for optimal results [6]. The application of HMMs for syscalls feature extraction was first introduced by Warrender et al [13], then extended by other authors [14–18].

The sequence models capture the semantic meaning of syscalls by calculating the probability distribution over the traces. These include but are not limited to Long-Short Term Memory (LSTM) [19], Gated Recurrent Units (GRUs), Recurrent Neural Networks (RNNs) [20], Word2Vec [21], and GloVe embeddings [22]. Sequence models have received much interest due to their remarkable ability in capturing inter-word correlations. Mikolov et al. proposed Word2Vec (W2V) [23, 24], a method that produces word vectors depending on their use context. Word vectors may be utilized in the Skip-Gram and Continuous Bag-of-Words models (CBOW). CBOW learns target words based on their context. The Skip-Gram model operates in the opposite direction, anticipating context from a target word. The fundamental concept underlying W2V is to train a neural network, discard the model, and then utilize the learned hidden layer weights as word vectors. Pennington et al. introduced Global Vectors for Word Representation, GloVe (GLV) [25], a count-based model that learns a context-sensitive vector representation of words. GLV views context as a co-occurrence matrix and incorporates word data accordingly. GLV is subsequently trained with the co-occurrence matrix’s non-zero elements.

These feature extractors all have advantages and peculiarities. Window-based feature extractors tend to require high computing costs for extracting features and model training, but they often result in increased detection rates. Although frequency-based techniques are less computationally expensive than window-based techniques, they do not necessarily produce high detection rates. Although training an HMM is a computationally expensive procedure, the approach frequently produces high detection metrics. W2V is simple and requires little to no preprocessing and low memory. GLV requires a lot of memory for storage because it is trained on the co-occurrence matrix, but the word vectors describe sub-linear correlations in the vector space, resulting in stronger models. In this paper, we specifically investigate W2V and GLV feature extraction techniques as they generate embeddings that provide implicit relationships, which are useful when training on data that can benefit from contextual information. The embeddings generated by W2V and GLV have been shown to improve generalization and performance for HIDS, especially when training data is scarce. We refer the interested reader to recent HIDS’ reviews presented by Liu et al. [26] and Bridges et al [6].

The work by Arp et al. [27] identifies ten common pitfalls of using AI applied to cybersecurity at the different stages of an ML workflow. Those pitfalls, however, do not include the discrepancies identified in this paper, which occur during the data processing/feature extraction stage. We demonstrate their impact as well as the simplest possible solution in the next section.

## Discrepancies in Feature Extraction Methods

In this work, we focus on the Australian Defense Force Academy Linux Dataset (ADFA-LD) [4, 28, 29], the Next-Generation Intrusion Detection System Dataset (NGIDS-DS) [30], the Web Conference 2019 Dataset (WWW2019) [31] and the Leipzig Intrusion Detection Dataset 2021 (LID-DS2021), an updated version of the LID-DS2019 [32]. We have selected these four datasets because they contain new and relevant intrusion types and were designed to assess the performance of modern HIDS. The ADFA-LD consists of 833 benign training, 4372 benign validation traces, and 746 attack training traces from six attack classes, namely, *user to root*, *password brute force* (FTP and SSH via the Hydra tool), *add new superuser*, *Java Based Meterpreter*, *Linux Meterpreter Payload*, and *C100 Webshell*. The NGIDS-DS comprises 19,256 benign and 18,121 attack traces and the WWW2019 dataset comprises 43,725 benign and 108,905 attack traces. The entire LID-DS2021 dataset is divided into train, validation, and test classes with recordings belonging to one of the four classes: idle, normal, attack, and the normal and attack combined. For experimental purposes, we only select the normal and attack recordings because they are the only classes of recordings or samples that appear in the other three datasets. We, therefore, combine all the partitions and present the original per-class binary compositions of the datasets in Table 1. The “Mean Length” column represents the mean length of all the traces in the dataset. In the ADFA-LD dataset, the shortest trace contains 76 syscalls and the longest contains 4495 syscalls. In the WWW2019 and NGIDS-DS datasets, the shortest traces contain only 1 syscall and the longest contain 349,986 and 471,177 syscalls, respectively. Traces in the LID-DS2021 are relatively longer than the ones in the other three datasets with the shortest trace containing 42 syscalls and the longest containing 10,698,062 syscalls.

**Table 1:** Composition of the datasets

Dataset	Total number of traces	Benign	Attack	Mean length
ADFA-LD	5951	5205	746	462.69
NGIDS-DS	37,377	19,256	18,121	2409.34
WWW2019	152,630	43,725	108,905	303.31
LID-DS2021	15,242	14,944	298	452261.96

W2V and GLV create embeddings for each call in the trace, therefore, to have fixed-size samples, most researchers take the average of all the embeddings of a trace as the final sample. We follow the same approach and use a standard value for the vector size $v=128$ for the W2V and GLV embeddings. Since we are dealing with sequences of numbers and not text, this approach introduces duplicates. This phenomenon is due to the nature of the samples and the vectorization technique used. An example is shown in Table 2, where we build a W2V model to generate embedding vectors of size $v=4$ for each trace. Although traces A and B, and C and D are different samples in the dataset, their embedding vectors of size 4, V1–V4, are the same for each pair (A–B and C–D) as we average the embedding vectors generated for each syscall. Having duplicate samples in the data is a significant research challenge in ML since it might lead to a data leakage scenario, in which the trained ML model is aware of a portion of the test data, that is, having data from the test data already present in the training data. One solution to this challenge involves keeping only one of these duplicated instances and removing the others. Nonetheless, this results in a loss of diversity and a lower sample size which may then lead to poor generalization.

**Table 2:** Toy example of duplicate system calls after vectorization with Word2Vec

Trace ID	Syscalls	Embedding vector			
		V1	V2	V3	V4
A	3 1 3	− 0.652	− 0.579	− 0.074	− 0.647
B	3 3 1 1 3 3	− 0.652	− 0.579	− 0.074	− 0.647
C	1 1 3 3	− 0.588	− 0.550	− 0.092	− 0.689
D	3 3 1 1	− 0.588	− 0.550	− 0.092	− 0.689

Table 3 shows the number of duplicates introduced by W2V and GLV in the four datasets. The “Benign” (“Attack”) column shows the initial number of benign (attack) samples in the dataset, and the “Duplicate Benign” (“Duplicate Attack”) column shows the number of duplicate benign (attack) samples after vectorization with the approach in the corresponding “Approach” column, the “Duplicate Attack & Benign” column shows the number samples that appear in both the benign and attack set after vectorization, and the “Final” column shows the final number of samples in the dataset after removing all the duplicates. In all the cases, except for the LID-DS2021 dataset, where the number of duplicated attack and benign samples is the same with both vectorization techniques, and the attacks in the ADFA-LD dataset, W2V has introduced more duplicates than GLV. As a result of deleting the duplicates, the final datasets contain fewer samples after vectorization with W2V.

**Table 3:** Quantitative descriptions after vectorization

Approach	Dataset	Benign	Duplicate benign	Attack	Duplicate attack	Duplicate attack and benign	Final
W2V	ADFA-LD	5205	2459	746	14	3	3475
	NGIDS-DS	19,256	7169	18,121	10,382	161	19,665
	WWW2019	43,725	12,353	108,905	20,027	45	120,205
	LID-DS2021	14,944	29	298	26	0	15,187
GLV	ADFA-LD	5205	2105	746	14	2	3830
	NGIDS-DS	19,256	5115	18,121	5764	116	26,382
	WWW2019	43,725	11,388	108,905	18,474	9	122,759
	LID-DS2021	14,944	29	298	26	0	15,187

To illustrate the influence of duplicated samples in the datasets, we constructed two ML models per dataset, one built on the data before the duplicated samples were removed and another constructed on the data after the duplicated samples were removed. Thus, we constructed a total of eight ML models with eight sets of evaluation metrics described in Sect. 3.1; and we present the preliminary results in Sect. 3.2.

### Experimental Setup

In the experiments conducted throughout the manuscript, we used the Extremely Randomized Trees (ERT) [33] as the binary classification algorithm to classify syscall traces as either “attack” or “benign”. The ERT is a tree-based ensemble method that builds multiple trees and splits nodes using random subsets of features. In the ERT, sampling is done without replacement, and the nodes are split into random splits [33]. This allows the ERT to be more computationally efficient than other tree-based ensemble algorithms that split on best splits such as Random Forests [34]. We set the number of trees parameter *t* of the ERT to $t = 1000$ and leave the other hyper-parameters at their default values.

In terms of performance assessment metrics, we considered the balanced accuracy, macro recall, macro precision, macro F_1_-score, and the Area Under the Receiver Operating Characteristic curve (AUROC). We constructed a robust stratified 5-fold cross-validation framework to report reliable metrics and account for potential sampling bias [27]. All experiments were conducted on the Digital Research Alliance of Canada (the Alliance), formerly Compute Canada, clusters using 4 NVIDIA A100 GPUs (each with a memory of 40 GB) and 48 CPU cores with 128GB of RAM.

### Preliminary Evaluation

**Fig. 1:**
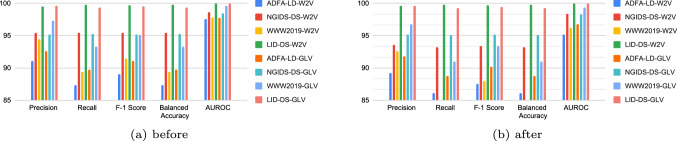
Performance of the ERT before (**a**) and after (**b**) removing the duplicates

Figure 1 shows the performance of the ERT before (Fig. 1a), and after (Fig. 1b) removing the duplicates introduced by W2V and GLV, respectively. The higher results depicted in Fig. 1a, are due to the potential inclusion of duplicate samples in the training and testing sets which are often overlooked by researchers [21, 35–37]; this, however, is not a correct form of evaluation, the correct results are those shown in Fig. 1b which appear to be lower.

**Fig. 2:**
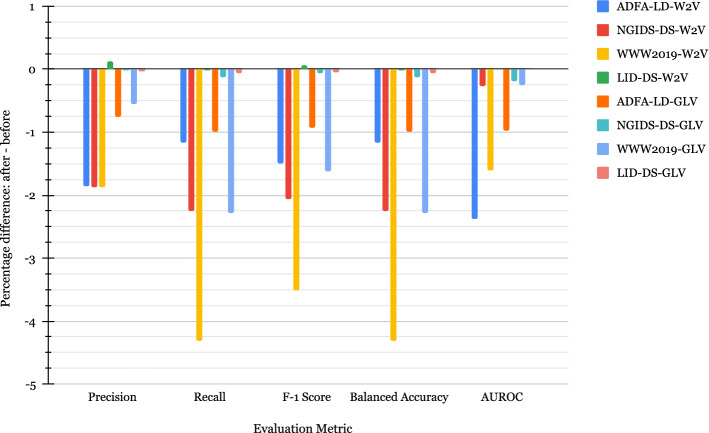
Difference in performance between after and before removing duplicates

Additionally, Fig. 2 shows the differences between the five pairs of metrics when the model is built after (Fig. 1b) and before (Fig. 1a) removing the replicated samples (*after–before*). Thus, a negative value shows that the model obtained without duplicates has a lower performance than the model trained with duplicates. It can be seen that with both feature sets, except on the LID-DS2021, we observe a drop in macro precision, recall, F_1_-score, and AUROC, on all the other datasets in the ranges 0.0193–4.3153% with the largest drop of 4.3153% in macro recall and balanced accuracy on the WWW2019 dataset with W2V embeddings. The LID-DS2021 dataset produces relatively stable results due to the fact that the vectorization techniques do not generate as many duplicates as they do for the other three datasets.

These findings highlight the need to find a more consistent feature set that will help reduce the number of duplicates and maintain data variety while also improving the performance of ML models. In the following experiments, we use the results from Fig. 1b as the baseline for comparisons, we will be referring to these feature sets as “*baseline W2V*” and “*baseline GLV*”.

## Experimental Analysis Using Alternate Feature Sets

**Fig. 3:**
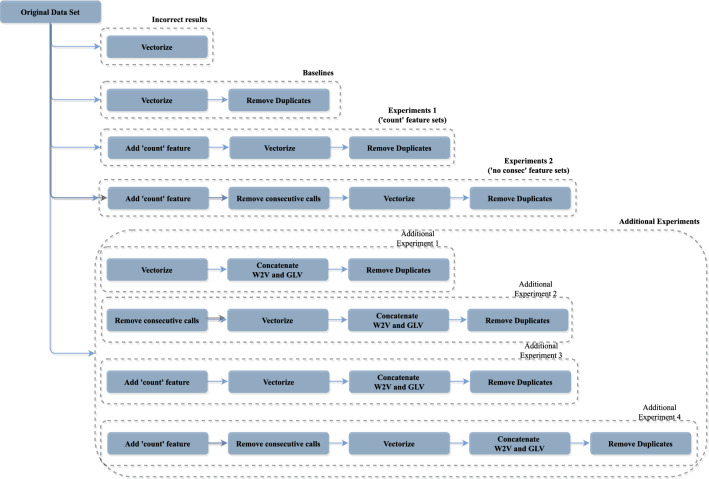
Experiments conducted in the paper

As mentioned in Sect. 3, using W2V and GLV vectorization techniques on these datasets introduces duplicated instances, leading to inaccurate results, especially when those duplicate embedding vectors appear in the training and testing partitions. Therefore, we remove the repeated samples and consider the models constructed with these vectors as our baselines. Figure 3 shows the experiments we conducted throughout the paper.

**Fig. 4:**
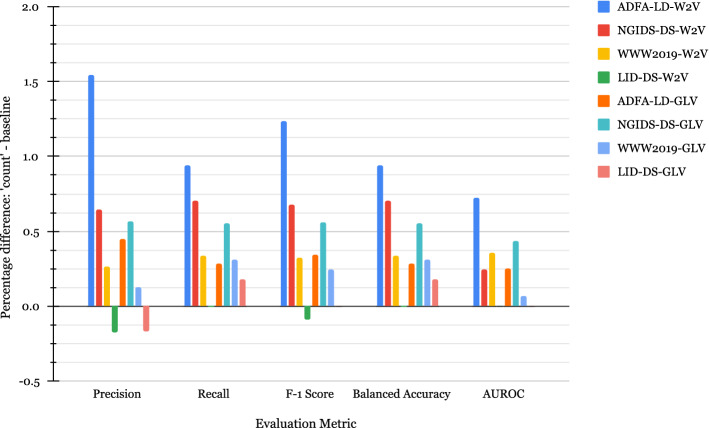
Difference in performance between “*baseline*” and “*count*” feature set

In our first set of experiments, we add a “*count*” feature to the embedding vectors of size 128 extracted for each system call trace. The “*count*” feature counts the total number of calls appearing in each trace to allow distinguishing between similar traces that may appear as duplicates after vectorization. The “*count*” column in Table 4 illustrates this new dimension. Although this new “*count*” feature does not help with traces C and D as they both contain the same number of system calls, it still helps to differentiate between traces A and B after vectorization, as we can observe in Table 4. Therefore, for each dataset, two feature sets of size 129 are extracted, to which we will be referring to as “*count W2V*” and “*count GLV*” to represent the usage of W2V and GLV, respectively. The quantitative descriptions of the vectorized datasets and the difference in performance metrics are presented in Table 5 and Fig. 4, while the respective evaluation metrics are shown in Fig. 8 in Appendix. As seen in Table 5, adding the “*count*” feature reduces the number of duplicates in all cases thus resulting in larger final sets compared to Table 3.

**Table 4:** Toy example of duplicate system calls and additional “*count*” feature

Trace ID	Syscalls	Embedding vector				*count*
		V1	V2	V3	V4	
A	3 1 3	− 0.652	− 0.579	− 0.074	− 0.647	3
B	3 3 1 1 3 3	− 0.652	− 0.579	− 0.074	− 0.647	6
C	1 1 3 3	− 0.588	− 0.550	− 0.092	− 0.689	4
D	3 3 1 1	− 0.588	− 0.550	− 0.092	− 0.689	4

**Table 5:** Quantitative descriptions after adding “*count*” feature

Approach	Dataset	Benign	Duplicate benign	Attack	Duplicate attack	Duplicate attack and benign	Final
W2V	ADFA-LD	5205	2433	746	14	2	3502
	NGIDS-DS	19,256	7161	18,121	10,381	162	19,673
	WWW2019	43,725	12,353	108,905	20,027	45	120,205
	LID-DS2021	14,944	23	298	25	0	15,194
GLV	ADFA-LD	5205	2105	746	14	2	3830
	NGIDS-DS	19,256	5101	18,121	5743	124	26,409
	WWW2019	43,725	11,388	108,905	18,474	9	122,759
	LID-DS2021	14,944	23	298	25	0	15,194

In Fig. 4, for all the datasets, we notice an increase in all the metrics compared to the baseline when building the model with both W2V and GLV embeddings. This indicates that adding the “*count*” feature is beneficial for these datasets when using W2V and GLV embeddings as it helps maintain diversity in the datasets.

**Fig. 5:**
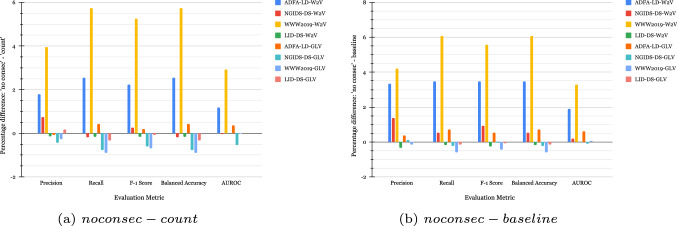
Difference in performance between “*no consec*” and “*count*” feature sets (**a**), and between “*no consec*” feature sets and “*baseline*” (**b**)

In our second set of experiments, we further remove the consecutive calls from a trace and only keep the first occurrence. Therefore, after applying this method, traces A and B, in Table 4, will have the exact same embedding vector, but the “*count*” dimension will prevent them from appearing as duplicates after vectorization. As traces are shorter, removing consecutive calls speeds up the vectorization process. Similar to our first experiment two feature sets of size 129 are generated for each dataset, we refer to those feature sets as “*no consec W2V*” and “*no consec GLV*”. The quantitative descriptions of the vectorized datasets and the evaluation metrics are presented in Table 6. This approach introduces fewer duplicate benign samples for W2V compared to Table 5, but increases the duplicate attacks samples and duplicate attack and benign samples in the two datasets expect for WWW2019. As for GLV, this approach increases the number of duplicate samples, except for the duplicate attack and benign samples in the NGIDS-DS dataset. Therefore, the final datasets obtained with GLV are smaller compared to the ones obtained with only the “*count*” feature in our previous experiment, but they are still larger than the ones obtained using W2V. Figure 5 shows the percentage differences between the “*no consec*” feature sets and the “*count*” feature sets (Fig. 5a), and between the “*no consec*” feature sets and the baselines (Fig. 5b). The respective evaluation metrics are shown in Fig. 9 in Appendix.

**Table 6:** Quantitative descriptions *without consecutive* calls and “*count*” feature

Approach	Dataset	Benign	Duplicate benign	Attack	Duplicate attack	Duplicate attack and benign	Final
W2V	ADFA-LD	5205	2401	746	30	8	3512
	NGIDS-DS	19,256	6572	18,121	12,539	183	18,083
	WWW2019	43,725	12,178	108,905	25,447	9	120,566
	LID-DS2021	14,944	27	298	16	0	15,199
GLV	ADFA-LD	5205	2224	746	30	6	3691
	NGIDS-DS	19,256	5727	18,121	10,502	121	21,027
	WWW2019	43,725	11,389	108,905	18,759	9	122,473
	LID-DS2021	14,944	13	298	14	0	15215

As seen in Fig. 5, on the ADFA-LD and WWW2019 datasets, we observe a significant increase in performance with the “*no consec W2V*” feature sets but no major change in performance with “*no consec GLV*” feature sets when compared to the “*baseline GLV*” and the “*count GLV*” feature sets. Similarly, on the NGIDS-DS and LID-DS2021 datasets, there is no significant increase or decrease in performance when compared to the baseline and “*count*” feature sets. This shows that removing the consecutive calls and keeping the “*count*” feature builds better models only when using W2V embeddings but not GLV embeddings.

In the consecutive experiments, whose results are shown in Fig. 10, we concatenate the embedding vectors created using W2V and GLV and build models with and without the “*count*” dimension added in the first experiment. This resulted in 4 new feature sets:

1. *256 w/ consec*: the feature set consisted of the combination of W2V and GLV embeddings with consecutive calls. This results from concatenating each of the feature sets used in the baseline, i.e., “*baseline W2V*” and “*baseline GLV*” (Fig. 1b).
2. *256 w/o consec*: the feature set consisted of the combination of W2V and GLV embeddings without consecutive calls.
3. *258 w/ consec*: the feature set consisted of the combination of W2V and GLV embeddings with consecutive calls and the “*count*” dimension added in the first experiment. This results from the concatenation of each of the feature set used in the first experiment, i.e., “*count W2V*” and “*count GLV*”.
4. *258 w/o consec*: the feature set consisted of the combination of W2V and GLV embeddings without consecutive calls but with the “*count*” dimension. This results from concatenating each of the feature sets used in the second experiment, i.e., “*no consec W2V*” and “*no consec GLV*”.

Due to the large number of features resulting from the concatenation of the W2V and GLV embeddings, dimensionality reduction or feature selection techniques should be applied to further minimize the number of features to only relevant features and reduce the computational complexity, speed up both training and detection, and possibly improve the model’s performance. This also helps avoid the curse of dimensionality [38, 39]. Feature selection, as the name implies, is simply preserving and eliminating specific features from the original dataset without changing them. On the other hand, dimensionality reduction discovers a smaller collection of new variables, each of which is a combination of the input variables and has the same information as the input variables. There exist several feature selection and dimensionality reduction methods. Fisher score [40], Backward (Forward) Feature Elimination (Selection) [41], and Missing Value Ratio are all examples of feature selection methods [42, 43]. Independent/Principal Component Analysis [44, 45], t-distributed Stochastic Neighbor Embedding (t-SNE) [46], Isometric Mapping (ISOMAP) [47], autoencoders [48] and Uniform Manifold Approximation and Projection (UMAP) [49] are examples of dimensionality reduction methods.

We therefore further conduct experiments using autoencoders for compressing or decompressing the features obtained by concatenating W2V and GLV embeddings. An autoencoder is a type of unsupervised Artificial Neural Network that compresses (or decompresses) data to a lower (or higher) dimension before reconstructing the input. The data representation in the lower (or higher) dimension is discovered by focusing on the relevant features and eliminating noise and redundancy. It employs an encoder-decoder architecture in which the encoder converts high-dimensional data to lower-dimensional data and the decoder attempts to recreate the original high-dimensional data from the lower-dimensional data.

**Fig. 6:**
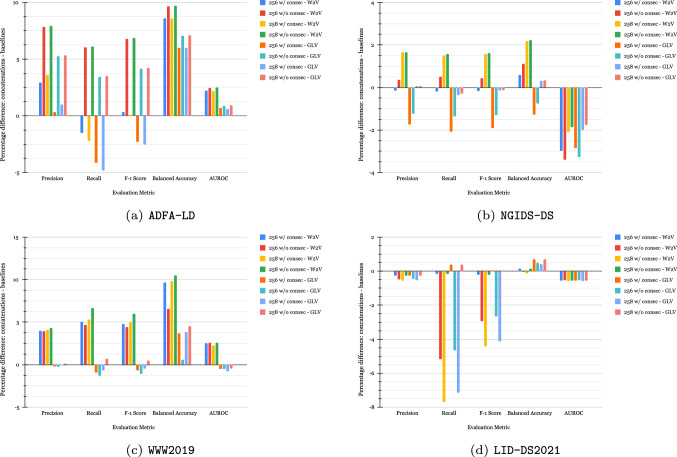
Difference in performance between the four feature sets and the W2V and GLV baselines on **a** ADFA-LD, **b** NGIDS-DS, **c** WWW2019 and **d** LID-DS2021 datasets

For each dataset and feature set, we build several autoencoder architectures and shapes and fine-tune them for the smallest reconstruction loss using the python package Hyperas available on GitHub.^1^ We report the best autoencoder architectures and parameters for each feature set in Table 8 in Appendix. Figure 6 shows the percentage differences between the four new feature sets and the baselines with W2V and GLV embeddings for each dataset, while we report the respective evaluation metrics and percentage differences with the other experiments in Figs. 10, 11 and 12 in Appendix.

As seen in Fig. 6, none of the four feature sets improve the AUROC when compared to the W2V and GLV baselines on the NGIDS-DS and the LID-DS2021. However, on the WWW2019, we notice an increase in all metrics when compared to baseline W2V but not GLV. We also observe that on the ADFA-LD dataset, the combinations without consecutive calls (“*w/o consec*”) yielded the highest results when compared to both W2V and GLV baselines. Nevertheless, the *count* dimension yielded the highest balanced accuracy, macro precision, recall and F_1_-score on the NGIDS-DS, while on the LID-DS2021 dataset, the feature set yielding the results on par with the baselines on all metrics was the one obtained from the concatenation of the original W2V and GLV embeddings without the “*count*” dimension (“*256 w/ consec*”), whereas the “*256 w/o consec*” and the “*258 w/ consec*” produced the poorest AUROC compared to both baselines.

Figure 13 in Appendix illustrates the ROC curves and mean AUROCs after stratified fivefold cross-validation with the four feature sets on the four datasets. It can be observed, in Fig. 13a–c, that when the FPR is in the range of 10–30%, all 4 feature sets achieve optimal TPRs on the ADFA-LD, NGIDS-DS and WWW2019 datasets with the “*258 w/o consec*” feature set yielding the highest AUROCs of 96.69%, 96.82%, and 98.84% with standard deviations of $\pm 0.45$, $\pm 0.23$ and $\pm 0.05$ respectively. As seen in Fig. 13d, on the LID-DS2021, optimal TPR is achieved much faster with the “*256 w/o consec*” feature set yielding an AUROC of 99.46% with a standard deviation of only $\pm 0.03$ on the LID-DS2021. It is worth noting that the “*258 w/o consec*” which yielded the highest AUROC on the other three datasets yielded the second highest AUROC on the LID-DS2021 dataset and the “*258 w/ consec*” yielded the lowest AUROCs on all datasets except for the NGIDS-DS.

## Analysis and Discussion

This section presents an analysis of the results from the experiments we conducted on the four datasets introduced in Sect. 3. On the ADFA-LD dataset, the “*256 w/o consec*” and “*258 w/o consec*” feature sets yielded the highest precisions (97.093% and 97.16%), recalls (94.31% and 94.38%), F_1_ (94.31% and 94.38%), balanced accuracies (95.79% and 95.85%) and AUROCs (97.65% and 97.69%). It should also be noted that the 4 new feature sets with the autoencoders yielded the lowest AUROCs compared to the other feature sets on the NGIDS-DS dataset, this may be due to the lack of benign samples in that specific set. On the NGIDS-DS dataset, the “*count GLV*” feature set yielded the highest precision, recall, F_1_, balanced accuracy and AUROC of 95.73%, 95.64%, 95.68%, 95.64%, and 98.71%. On the WWW2019 dataset, on the other hand, the “*258 w/o consec*” feature set yielded the highest precision, recall, F_1_, and balanced accuracy of 96.85%, 91.72%, 93.92% and 95.52% while the “*no consec W2V*” feature set yielded the highest AUROC of 99.53%. On the LID-DS2021 dataset, the “*no consec GLV*” feature set yielded the highest precision of 99.63%, the “*256 w/ consec*” and the “*258 w/o consec*” feature sets yielded the best accuracies (99.96%), the “*count W2V*” feature set yielded the highest AUROC of 99.9998% but none of the features and approaches outperformed the baseline in terms of F_1_ and recall with the “*baseline W2V*” feature set yielding the highest F_1_ of 99.71% and recall of 99.8081%.

These results show that despite not outperforming the baselines on all metrics on the LID-DS2021 datasets, the new feature sets increase the performance of the ML models relative to the baselines, especially on the other three datasets where the “*258 w/o consec*” feature set has yielded relatively high results. In addition to the performance improvement, they help reduce the number of duplicated samples and maintain data diversity, as stated in Sect. 4.

We further performed the Friedman test [50, 51], which is the non-parametric equivalent of the repeated-measure Analysis Of Variance (ANOVA) for statistical hypothesis testing and determining whether there are any significant differences among the measures obtained in our experiments Fig. 3. Table 7 provides the *Q*-statistics, *p*-values, and Critical Distances (CD) of the four groups of evaluation metrics yielded by the ten feature sets, i.e., the baselines (Fig. 1b), the added count dimensions (“*count*” feature sets, Fig. 8), the added count dimension without the consecutive calls (“*no consec*” feature sets, Fig. 9) and the four combinations (Fig. 10) on the four experimental datasets. We can see that the *Q*-statistics for the ADFA-LD, NGIDS-DS, WWW2019, and LID-DS2021 datasets come out to be equal to 33.82909, 31.77818, 34.17818, and 23.40881 and the respective *p*-values are 0.00009, 0.00021, 0.00008 and 0.00534. Since these *p*-values are all less than 0.05 at our confidence level of 0.95, we can reject the null hypothesis that the obtained metrics are the same for all ten groups of feature sets. In simple words, we have enough proof to say that the differences among the ten feature sets’ results are statistically significant.

**Table 7:** *Q*-statistic, *p*-value and critical distance

Dataset	${Q}$	*p*-value	CD
ADFA-LD	33.82909	0.00009	4.28364
NGIDS-DS	31.77818	0.00021	4.28364
WWW2019	34.17818	0.00008	4.28364
LID-DS2021	23.40881	0.00534	4.28364

**Fig. 7:**
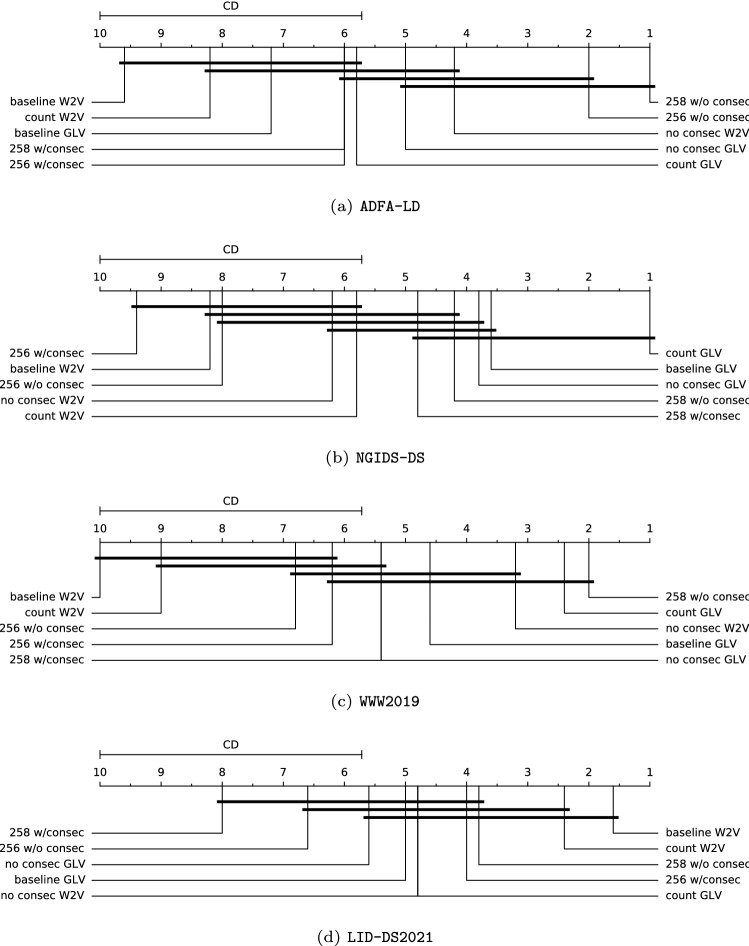
CD diagrams of the ten feature sets on **a** ADFA-LD, **b** NGIDS-DS, **c** WWW2019, and **d** LID-DS2021 datasets

**Fig. 8:**
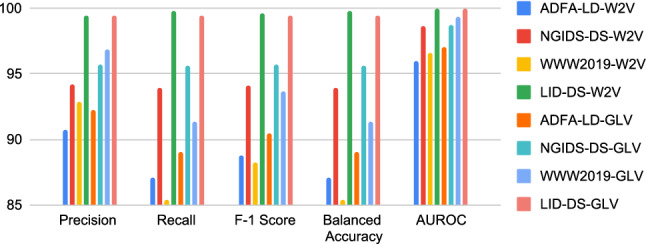
Performance of the ERT with “*count*” feature (*count* feature set)

**Fig. 9:**
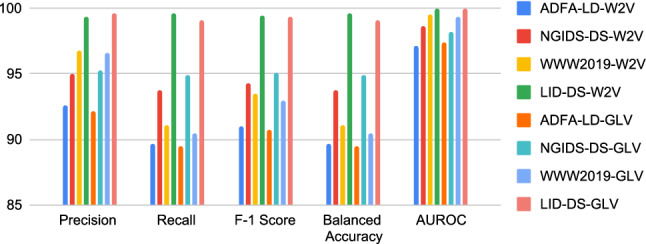
Performance of the ERT *without consecutive* calls and “*count*” feature (*no consec* feature set)

**Fig. 10:**
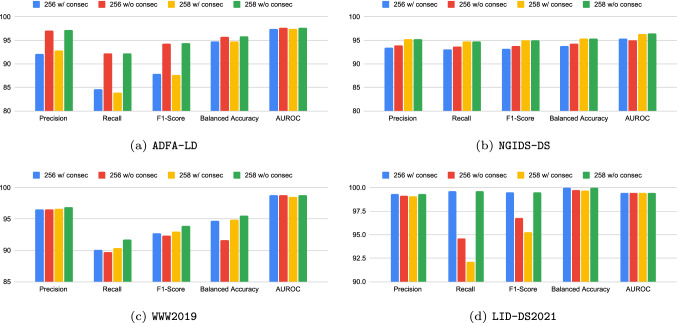
Performance of combining Autoencoders and ERT on **a** ADFA-LD, **b** NGIDS-DS, **c** WWW2019 and **d** LID-DS2021 datasets using the 4 new feature sets

**Fig. 11:**
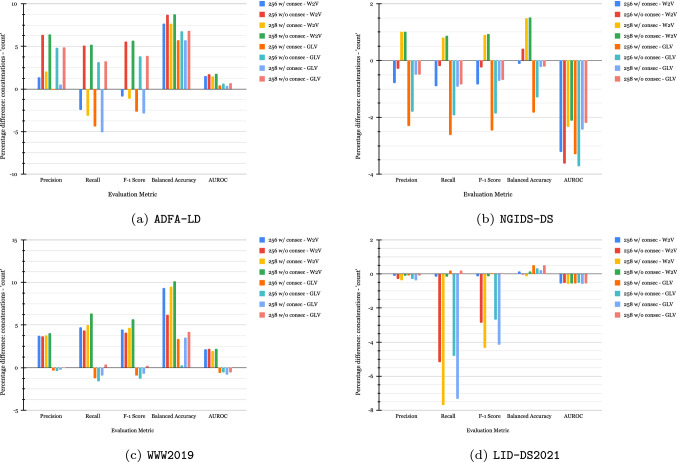
Difference in performance between the four feature sets and the W2V and GLV “*count*” feature sets on **a** ADFA-LD, **b** NGIDS-DS, **c** WWW2019 and **d** LID-DS2021 datasets

**Fig. 12:**
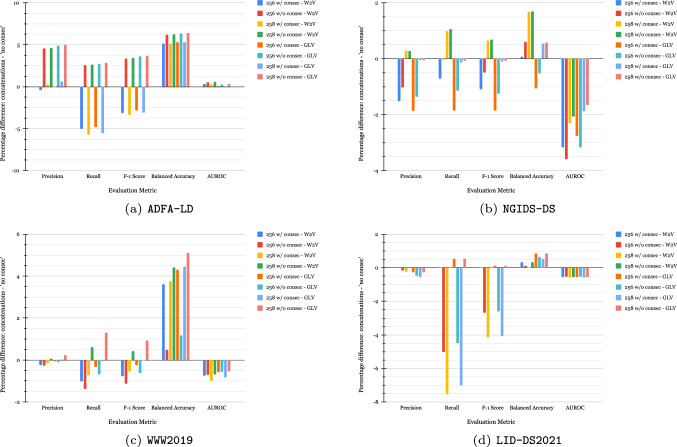
Difference in performance between the four feature sets and the W2V and GLV “*no consec*” feature sets on **a** ADFA-LD, **b** NGIDS-DS, **c** WWW2019 and **d** LID-DS2021 datasets

**Fig. 13:**
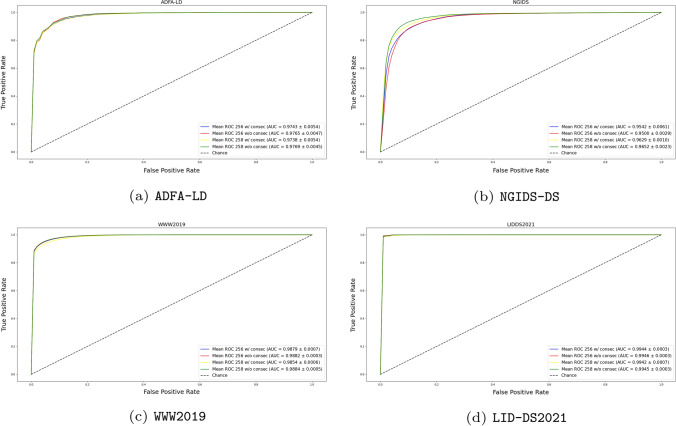
ROC curves of the combination of autoencoders and ERT on **a** ADFA-LD, **b** NGIDS-DS, **c** WWW2019 and (d) LID-DS2021 datasets

We, therefore, conducted the Nemenyi test [52, 53] to find precisely which feature sets have different means. As seen in Table 7, the CD is 4.28364 for all four datasets, in other words, there is a statistically significant difference between the feature sets if their average ranks differ by at least 4.28364. These comparisons are depicted on critical distance diagrams (Fig. 7). A connecting line between feature sets indicates that the null hypothesis that they are significantly different cannot be rejected. On the ADFA-LD dataset, Fig. 7a, we observe that the “*258 w/o consec*” feature set ranked first and the “*256 w/o consec*” ranked second. This shows that concatenating the two feature sets without consecutive provides better rankings overall on this dataset. In Fig. 7b, the “*count GLV*” feature set ranked first on the NGIDS-DS which was followed by the “*baseline GLV*” and “*no consec GLV*”. It can be inferred that the GLV embeddings provide better overall rankings for this dataset. Similar to the ADFA-LD, Fig. 7c shows that the “*258 w/o consec*” also ranked first on the WWW2019 along with the “*count GLV*” feature set which also ranked first on the NGIDS-DS. The “*baseline W2V*” feature set ranked first on the LID-DS2021 and the “*count W2V*” ranked second (Fig. 7d). This is the only dataset in which the baseline ranked first. We posit that this is because the number of replicas was already low, to begin with, thus the extra features had no large impact on the performance of the models. The “*258 w/o consec*” feature set ranked first on both the ADFA-LD and WWW2019 datasets, the “*count GLV*” feature set ranked first on the NGIDS-DS and second on the WWW2019; the *baseline W2V* feature set ranked first on the LID-DS2021 dataset and second on the NGIDS-DS. These results demonstrate that by using the new feature sets, it is possible to preserve data variety, reduce the number of redundant samples, and achieve high performance.

## Conclusion

In this paper, we have demonstrated how word-embedding-based feature extraction techniques, namely W2V and GLV, can lead to a loss of diversity and the introduction of replicas in the data from Host-Based Intrusion Detectors. These issues are frequently overlooked by researchers. However, the replicated instances can end up appearing in both the training and testing sets, leading ML models to provide overly optimistic and inaccurate results, rendering the ML-based Intrusion Detector ineffective. We have used the ADFA-LD, NGIDS-DS, WWW2019, and LID-DS2021 datasets for experimentation and demonstrated that W2V introduces more replicated samples than GLV, which results in a greater performance decline when those samples are removed. We have therefore conducted extensive experiments using alternate feature sets that consisted of adding new dimensions and concatenating the features extracted by W2V and GLV to simultaneously tackle the issue of duplicated samples and improve the performance of the models. Experimental results show that, from the ten feature sets, concatenating embeddings from W2V and GLV and counting the number of syscalls in a trace could increase the performance of the ERT in three out of the four datasets. Nonetheless, there is no universally suitable feature set that can be used across all the experimental datasets as they have all yielded different results on the datasets. Selecting the ideal feature set for a specific dataset should therefore be based on the user’s preference. Finally, as seen in Table 4, our proposed feature sets are still limited in the sense that duplicate traces may still appear in the datasets even with the alternate feature sets. Future work will include addressing the duplicate samples that our feature sets were unable to handle, generating a universally applicable feature set, evaluating our feature sets on additional HIDS datasets and looking into data augmentation techniques to solve the data imbalance issue. An additional research direction would be evaluating our proposed feature sets on multi-class scenarios.

## Data Availability

The datasets analyzed during the current study are available from the corresponding author on reasonable request.

## Appendix: Performance Metrics and Parameters

### Additional Performance Metrics Results

This section shows the different metrics yielded by the ERT on the additional feature sets. Figure 8 shows the performance of the ERT with the “*count*” feature set added in Experiments 1. Figure 9 shows the performance of the ERT with the “*no consec*” feature set built in Experiments 2. Figure 10 shows the respective performance metrics for each dataset using the four feature sets built in Additional Experiments. Figures 11 and 12 show the percentage differences between the four new feature sets and the “*count*” and “*no consec*” feature sets respectively.

### Best Parameters of Different Auto-Encoder Architectures

Table 8, shows the best autoencoder architectures and parameters from Hyperas that we used to compress/decompress the data before building the intrusion detection models we evaluated in Fig. 10. The hidden neurons column represents the neurons after (before) the input (output) layers which are 256 or 258 depending on the feature set.

**Table 8:** Best autoencoder architecture per feature set

Feature set	Activation	Alpha	Batch_size	Dropout	h_n	l2	Optimizer
ADFA256_consec	sigmoid	0.7623	256	0.1576	[128, 64, 128]	2e−06	rmsprop
ADFA256_noconsec	relu	0.3841	8	0.0738	[128, 64, 32, 16, 8, 16, 32, 64, 128]	2e−02	adam
ADFA258_consec	relu	0.2222	16	0.2549	[512, 1024, 1024, 512]	5e−05	rmsprop
ADFA258_noconsec	sigmoid	0.9524	8	0.5507	[128, 64, 32, 16, 8, 8, 16, 32, 64, 128]	2e−02	sgd
WWW256_consec	tanh	0.6267	256	0.0167	[224, 192, 160, 128, 160, 192, 224]	5e−07	adam
WWW256_noconsec	tanh	0.6267	256	0.0167	[224, 192, 160, 128, 160, 192, 224]	1e−05	adam
WWW258_consec	tanh	0.6267	256	0.0167	[224, 192, 160, 128, 160, 192, 224]	1e−05	adam
WWW258_noconsec	tanh	0.6267	256	0.0167	[224, 192, 160, 128, 160, 192, 224]	1e−05	adam
NGIDS256_consec	tanh	0.2181	256	0.7916	[128, 64,32,32, 64, 128]	1e−05	adam
NGIDS258_consec	tanh	0.6267	256	0.0167	[224, 192, 160, 128, 160, 192, 224	2e−05	rmsprop
NGIDS256_noconsec	sigmoid	0.9028	16	0.2094	[256, 512, 1024, 1024, 512, 256]	3e−05	adam
NGIDS258_noconsec	sigmoid	0.9028	16	0.2094	[256, 512, 1024, 1024, 512, 256	3e−05	adam
LIDDS256_consec	tanh	0.3346	64	0.4032	[2048,1024,512, 1024, 1024,2048]	1e−04	rmsprop
LIDDS258_consec	tanh	0.9387	32	0.4577	[1024, 1024, 1024]	2e−05	sgd
LIDDS256_noconsec	tanh	0.5799	128	0.6749	[1024, 2048, 1024]	2e−04	adam
LIDDS258_noconsec	tanh	0.6300	256	0.1742	[512, 1024, 2048, 1024, 512]	4e−06	adam

h_n: hidden neurons

## Footnotes

1 https://github.com/maxpumperla/hyperas.
